# Investigations into the impact of various substrates and ZnO ultra thin seed layers prepared by atomic layer deposition on growth of ZnO nanowire array

**DOI:** 10.1186/1556-276X-7-368

**Published:** 2012-07-03

**Authors:** JN Ding, YB Liu, CB Tan, NY Yuan

**Affiliations:** 1 Center for Low-Dimensional Materials, Micro-Nano Devices and System, Changzhou University, Changzhou, 213164, China; 2 Jiangsu Key Laboratory for Solar Cell Materials and Technolgy, Changzhou, 213164, China

**Keywords:** ZnO, Seed layers, The fluctuate amplitude, Frequency of roughness

## Abstract

The impact of various substrates and zinc oxide (ZnO) ultra thin seed layers prepared by atomic layer deposition on the geometric morphology of subsequent ZnO nanowire arrays (NWs) fabricated by the hydrothermal method was investigated. The investigated substrates included B-doped ZnO films, indium tin oxide films, single crystal silicon (111), and glass sheets. Scanning electron microscopy and X-ray diffraction measurements revealed that the geometry and aligment of the NWs were controlled by surface topography of the substrates and thickness of the ZnO seed layers, respectively. According to atomic force microscopy data, we suggest that the substrate, fluctuate amplitude and fluctuate frequency of roughness on ZnO seed layers have a great impact on the alignment of the resulting NWs, whereas the influence of the seed layers' texture was negligible.

## Background

Zinc oxide (ZnO) is a semiconductor with wide band-gap (3.37 eV) and possesses high excited binding energy of 60 meV. [1,2] It has been widely studied and applied to field effect transistors [3], field emitters [4], photodetectors [5], gas sensors [6], dye-sensitized solar cells [7], and other optoelectronic devices [8,9] because of its special properties, such as long-term stability, relatively low material costs, simple processing due to its compatibility with wet chemical etching, biocompatibility, environmental friendliness, excellent radiation resistance, and so on. In these applications, one-dimensional (1D) and nanoscale ZnO materials (e.g., nanorods, nanowires, and nanotubes) have attracted considerable attention due to their significantly different electronic and photoelectron-chemical properties and have potential applications in electronic and photonic devices [10-14].

To obtain 1D ZnO materials, there are several methods including physical vapor phase growth that required high temperature and chemical approaches working at low temperature, in which hydrothermal synthesis is a good chemical approach for the synthesis of ZnO nanowire arrays (NWs) by fabricating ZnO seeds with the morphology of thin films or nanoparticles on substrates firstly [15,16]. Atomic layer deposition (ALD) is a good method for growing high-quality ZnO seed layers [17-20] because it requires low growth temperature and can offer excellent conformality, easy and accurate thickness control, good reproducibility, and high uniformity over a large area. However, the reported thickness of the seed layers prepared by ALD was greater than 10 nm [19,20]. Moreover, although the effect of roughness and texture of seed layers on the alignment of NWs has been reported [19,21,22], few research focused on the mechanism on how the roughness of seed layers affected the orientation of NWs.

## Presentation of the hypothesis

In this paper, we first deposited ZnO seed layers with different thickness (2 to 50 nm) on different substrates by ALD method. The effect of the substrates and the seed layers' thickness on morphology and alignment of subsequent ZnO nanorods prepared by hydrothemal method was studied. It was found that roughness rather than the texture of the ZnO seed layers had a great impact on the alignment of the resulting NWs.

## Testing the hypothesis

In this article, the ALD technique was employed to deposit ZnO seed layers on various substrates. The substrates included B-doped ZnO (BZO) films, indium tin oxides (ITO) films, single crystal (111) silicon, and glass sheets. Diethylzinc [DEZ, Zn(C_2_H_5_)_2_] and deionized water were used as the precursors for ZnO deposition. Pure N_2_ gas (99.999%) was used to carry and purge gas. The reaction is carried out as follows:

(1) $$Zn{\left(C_{2}H_{5}\right)}_{2}+H_{2}O\to ZnO+2C_{2}H_{6}$$

The reaction chamber was pumped down to 1 to 2 Torr before deposition. The operating environment of ZnO deposition was maintained at 3 Torr and 200°C. Each deposition cycle consisted of four steps, which included DEZ reactant, N_2_ purge, H_2_O reactant, and N_2_ purge. The typical pulse time for introducing DEZ and H_2_O precursors was 0.5 s, and the N_2_ purge time was 10 s. The deposition cycles of 11, 22, 33, 44, 55, 110, and 275 were chosen to produce ZnO seed layers with the various thickness of 2, 4, 6, 8, 10, 20, and 50 nm. The deposition rate at the above conditions approaches 0.182 nm/cycle.

The subsequent hydrothermal growth was carried out at 90°C in a sealed kettle by immersing the deposited substrates in aqueous solution (80 mL) containing zinc nitrate (Zn(NO_3_)_2_·6H_2_O, 0.01 mol/L) and hexamethylenetetramine (HMTA; C_6_H_12_N_4_ 0.01 mol/L). ZnO NWs were fabricated according to the following reactions.

(2) $${\left(CH_{2}\right)}_{6}N_{4}+6H_{2}O\to 6HCHO+4NH_{3}$$

(3) $$NH_{3}+H_{2}O\to NH_{4}{}^{+}+OH^{-}$$

(4) $$2OH^{-}+Zn^{2+}nO(s)+H_{2}O$$

Finally, the samples were washed with deionized water and dried in air before characterization. The morphology of the NWs was characterized by scanning electron microscopy (SEM, Philips FEIXL30 SFEG, Amsterdam, Netherlands) and transmission electron microscopy (TEM, Hitachi HF-2000, Chiyoda, Tokyo, Japan). TEM samples were prepared by gently dragging the holey (400 mesh Cu, SPI supplies, West Chester, PA, USA) carbon grids along the surface of the samples. X-ray diffraction (XRD) analysis was performed with a Rigaku Dmax-2000 diffractometer using CuKa radiation (Rigaku Corporation, Tokyo, Japan). The morphology of the seed layers and roughness was characterized by an atomic force microscope (AFM, Park Systems XE-100, Santa Clara, California, USA). The photoluminescence (PL) spectroscopy is performed on an Olympus BX51 microscope with Hg illumination and UV filter cube (U-MWU2, excitation, Olympus Shinjuku, Tokyo, Japan).

**Figure 1:**
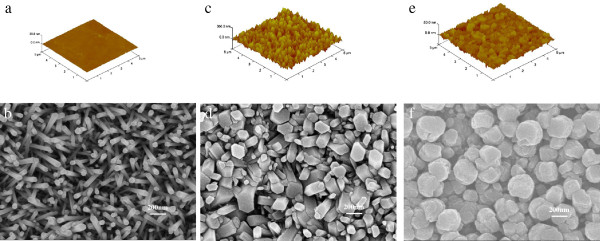
**AFM three-dimenional images.** AFM three-dimensional images of (**a**) Si substrates, (**c**) BZO substrates, and (**e**) ITO substrates SEM images of NWs grown on Si substrates (**b**), on BZO substrates and (**d**), on ITO substrates (**f**).

Figure 1b shows the typical hexagonal cylinder shaped ZnO NWs grown on Si and glass substrates with the 10-nm-thick ZnO seed layers. The morphology is different from that of the NWs grown on the BZO (Figure 1d) and ITO substrates (Figure 1f) although the thickness of the seed layers is the same of 10 nm. Figure 1d shows the inclined NWs grown on the BZO substrate that have short and thick geometry morphologies. The diameters of the NWs range from several tens of nanometers to hundreds of nanometers. It is noteworthy that the diameters of the NWs are consisted with the size of the grains on the surface of BZO film (shown in Figure 1c). Analogously, the nonuniform rough ITO surface with the several tens of nanometers grain size (shown in Figure 1e) produced anomalous NWs with the average diameter of about 200 nm. Different from the Si and glass substrates, the BZO and ITO films have obvious grain boundaries on the film surfaces. Grains can be the site of nucleate for the growth of NWs and ZnO seed layer with a 10 nm thickness is too thin to shield the morphologies of BZO and ITO films. So the morphologies of BZO and ITO films have a great influence on NWs, which results in the NWs having similar geometric morphology with the substrate surface. So we get the conclusion that the NWs prepared by hydrothermal reaction were influenced greatly by surface topography of substrates when substrates are covered with ultra-thin seed layers.

**Figure 2:**
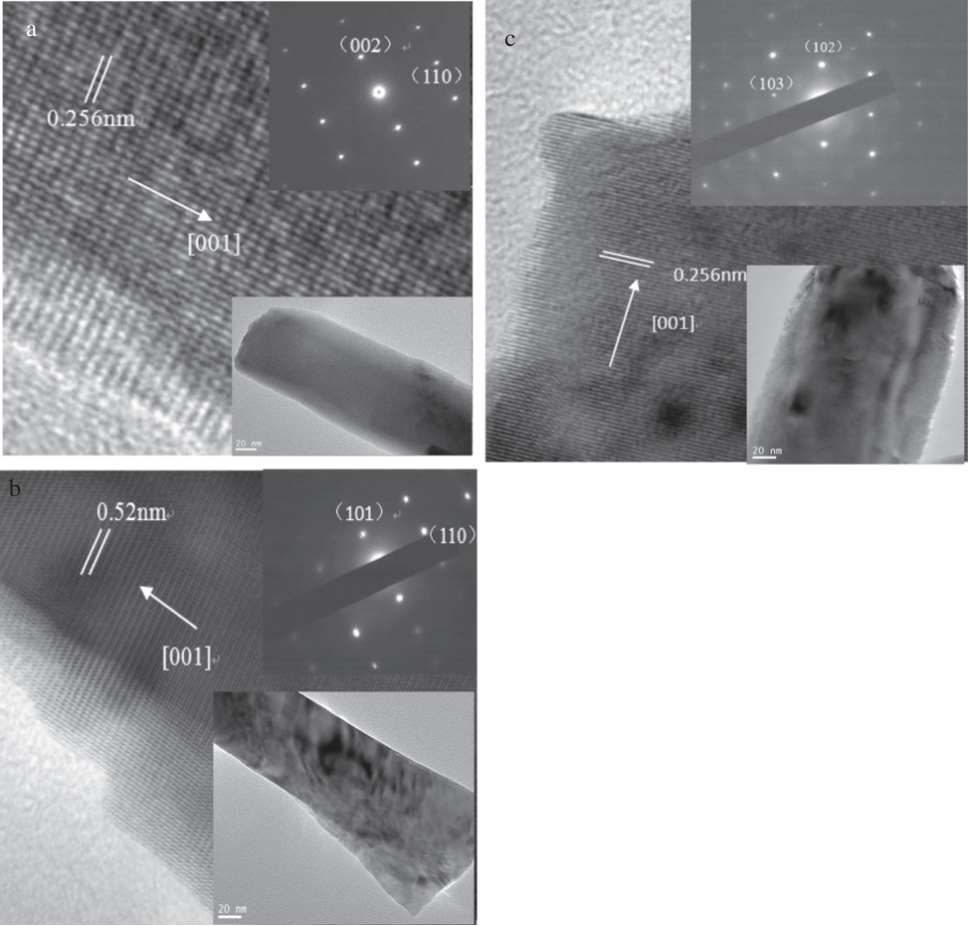
**The high- and low-resolution images and the selected area diffraction patters.** High-resolution TEM images, low-resolution TEM images, and the selected area electron diffraction patterns (see inset) of NWs grown on Si substrate (**a**), on BZO substrate and (**b**), on ITO substrate (**c**).

To learn more about these ZnO NWs, TEM was used to characterize the ZnO NW structures. Figure 2 shows high-resolution TEM images taken from ZnO NWs grown on various substrates. The insert figures show the corresponding low-resolution TEM images and selected-area electron diffraction patterns, which indicates that the ZnO nanorods are single-crystalline in structure. The HRTEM images of ZnO nanorods grown on various substrates reveal clear lattice spacing of 0.52 or 0.25 nm correspond to the inter-planar spacing of the wurtzite ZnO (001) or (002) face, which indicate that the ZnO nanorod growth occurs preferentially along the [001] direction. So the crystal structures of NWs prepared by hydrothermal reaction were not influenced by surface topography of substrates.

**Figure 3:**
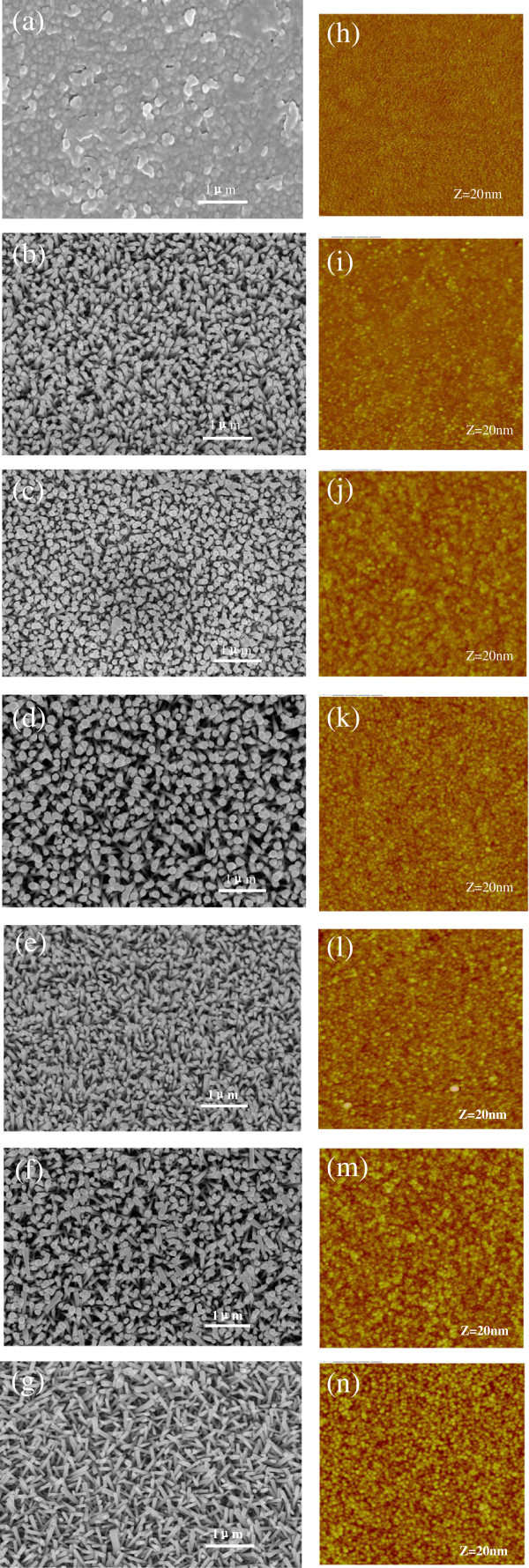
**SEM images of the NWs grown on ZnO seed layers with different thickness.** (**a**) 2 nm, (**b**) 4 nm, (**c**) 6 nm, (**d**) 8 nm, (**e**) 10 nm, (**f**) 20 nm, (**g**) 50 nm and AFM images of ZnO seed layers with different thickness (**h**) 2 nm, (**i**) 4 nm, (**j**) 6 nm, (**k**) 8 nm, (**l**) 10 nm, (**m**) 20 nm, and (**n**) 50 nm. The lateral scan dimensions are 2 μm × 2 μmm, and the Z value denotes the full vertical length scale.

In order to understand the relationship between the thin seed layer and the NWs, a more systematic structural investigation was carried out. Figure 3 shows SEM photographs of NWs grown on glass substrates pre-coated with ZnO seed layers. The ZnO seed layers with thickness from 2 to 50 nm were deposited by ALD method. It can be clearly found that ZnO nanoparticles grow out of the 2-nm-thick seed layer, whereas NWs grow out of the seed layers whose thickness is above 4 nm (Figure 3a). Moreover, the NWs on 6- and 8-nm-thick seed layers have the best alignment, with an average rod diameter of 100 nm (Figure 3c,d). However, the relatively sparse and poorly aligned NWs are obtained on the seed layers with the thickness greater than 10 nm, and their orientation gets worse and worse with the increase of the seed layers' thickness (Figure 3e,f,g). For example, the NWs on the 20-nm-thick seed layer are more disordered than those on the seed layers with the thickness of 6 and 8 nm. When the seed layer's thickness reaches 50 nm, almost all the NWs are slanting as shown in Figure 3g. Therefore, the threshold thickness of the seed layers for the conversion between the well-aligned and poorly-aligned NWs is 8 nm.

**Figure 4:**
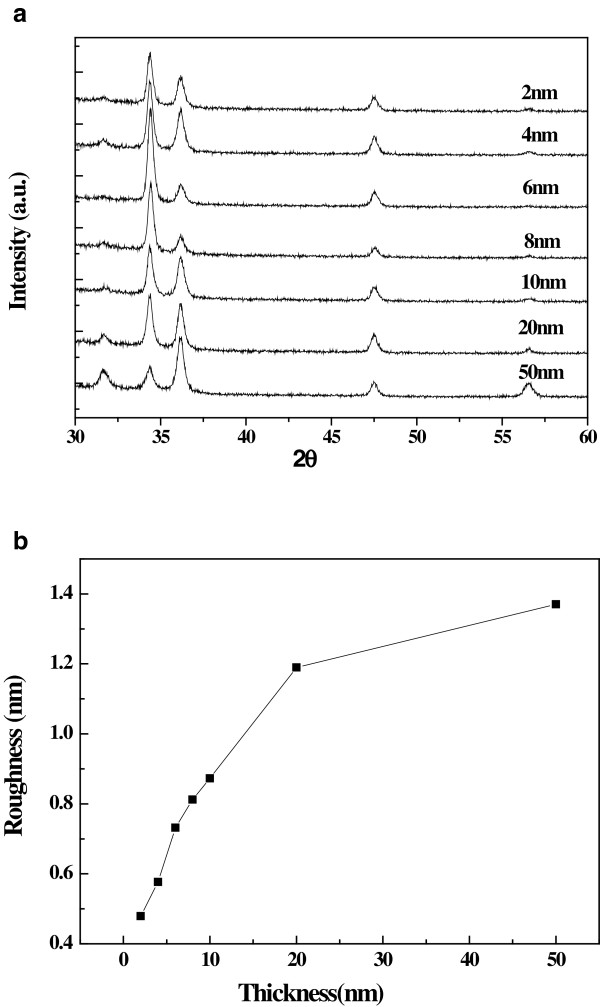
**The XRD spectra and ZNO peak ratios.** (**a**) XRD spectra of NWs grown on pre-coated substrates with different thickness (from top to bottom, 2, 4, 6, 8, 10, 20, and 50 nm). (**b**) ZnO peak ratios for (1 0 1) to (0 0 2) as a function of substrate thickness.

In Figure 4a, the crystal structure of the NWs was examined. All the diffraction peaks can be indexed to the wurtzite structure of ZnO (36–1451). The peak intensity ratio of (101) to (002) according to the different thickness of the seed layers is shown in Figure 4b. As discussed above, the NWs grown on the seed layer with the thickness of 6 or 8 nm exhibit a strong peak intensity ratio, indicating good orientation of the NWs. Consequently, the XRD results are consistent with SEM results in Figure 3. It should be noted that the peak intensity ratio of the nanoparticles grown on the 2-nm-thick seed layer exhibits the third largest value, which indicates that the nanoparticles prefer the growth along the c-axis direction even if it is a failure to generate NWs due to very thin seed layer.

**Figure 5:**
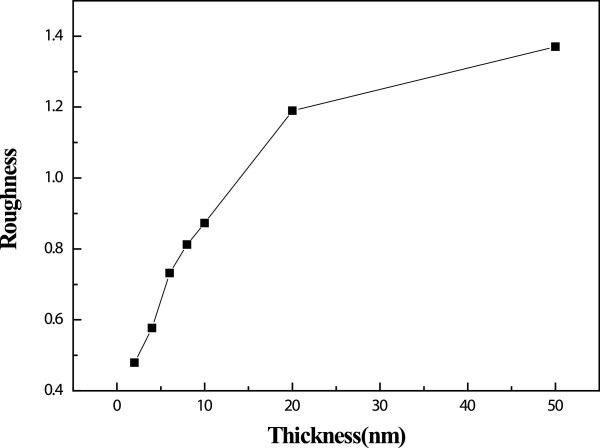
The dependence of roughness on seed thickness.

We suggest that one of the important reasons for the alignment variation according to different thickness of the seed layers is the ZnO seed roughness, which is also reported by previous research [19,21]. The images of the ZnO seed films with different thickness deposited on glass substrates were characterized by AFM. As shown in Figure 5, their roughness increases from 0.479 to 1.37 nm with their thickness (from 2 to 50 nm).

**Figure 6:**
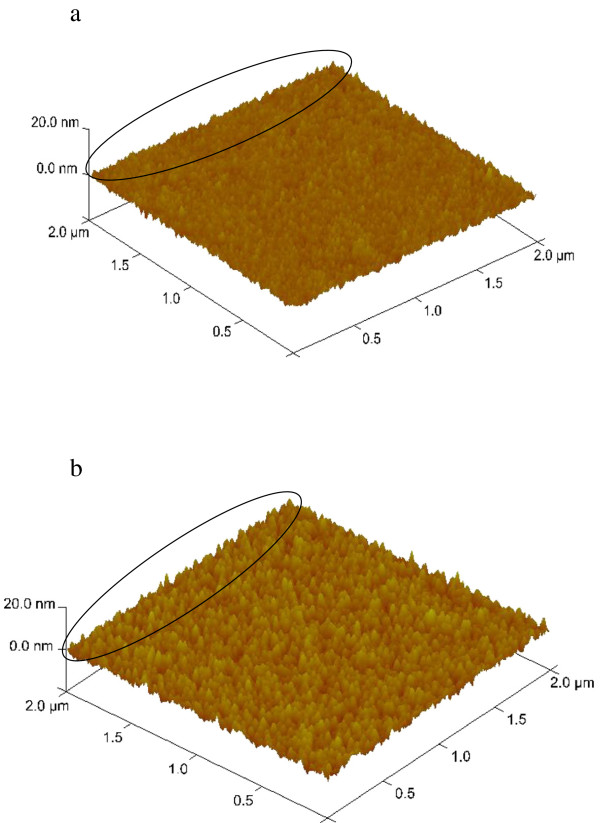
AFM three-dimensional images of (a) 6 nm seed layers and (b) 50 nm seed layers.

**Figure 7:**
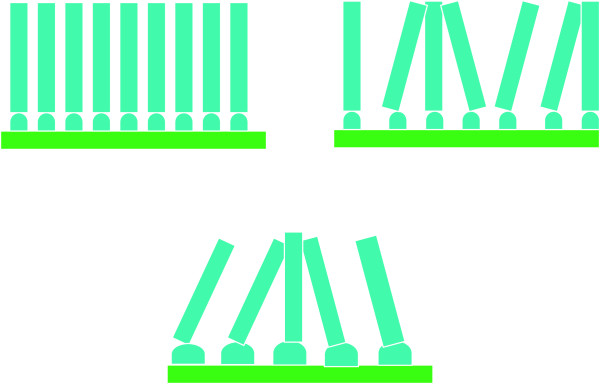
**The schematic model.** Schematic model for the effect of fluctuate amplitude and frequency of seed layer roughness on the alignment of NWs.

The reason why roughness affects orientation of NWs has been hypothesized and proved. It is well known that roughness represents fluctuation amplitude and frequency of substrate or film surface, which plays an important role in nucleation and growth of NWs [21]. However, the fluctuation amplitude and frequency of roughness also determine the orientation. Figure 6 shows three-dimensional images of the seed layers with the thickness of 6 and 50 nm. As shown in the edges indicated by the circle in Figure 6, it could be found that compared with that on 6-nm-thick seed layer, the fluctuation amplitude and frequency of the roughness for the 50-nm-thick seed layer are larger and smaller than those for the 6-nm-thick seed layer, respectively, which may be caused by stack of the ZnO nanoparticles. The augment of spacing of local peaks weakens interaction among ZnO nanorods, which leads to free growth and slant of some nanorods. This relationship is shown schematically in Figure 7.

**Figure 8:**
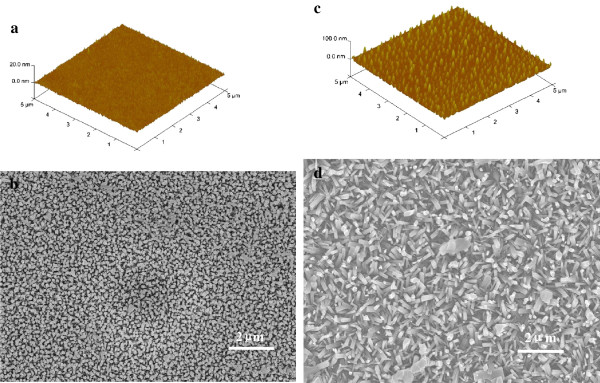
**AFM and SEM images.** (**a**)AFM image of 6-nm-thick seed layer. (**b**)SEM image of NWs grown on 6-nm-thick seed layer. (**c**) AFM image of 6-nm-thick seed layer after annealing. (**d**)SEM image of NWs grown on annealed 6-nm-thick seed layer.

Another convincing evidence that the fluctuation amplitude and frequency of roughness affect orientation of NWs is shown in Figure 8. Figure 8a, c gives AFM photos of 6-nm-thick seed layers before and after annealing. Comparing Figure 8b,d, it can be found that the alignment of NWs obtained on the annealed seed layer becomes poor. Although annealing usually can improve the crystallinity of the seeds, the peak spacing of the seed layers increases after annealing, resulting in poor alignment of the NWs. This result shows that the fluctuation amplitude and frequency of roughness determine the orientation.

**Figure 9:**
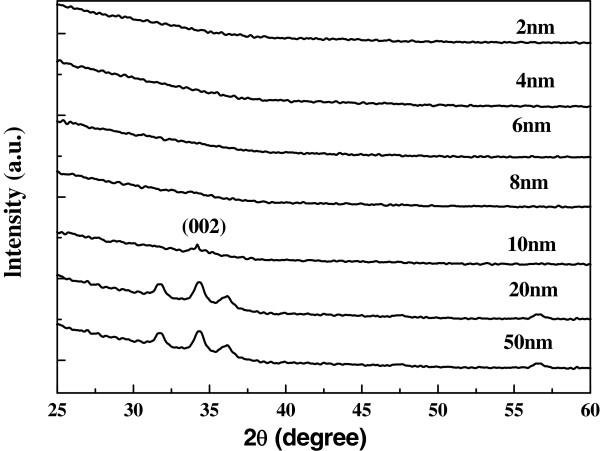
**XRD spectra of ZnO seed layers.** The XRD spectra of ZnO seed layers with different thickness (from top to bottom, 2, 4, 6, 8,10, 20, and 50 nm).

The texture of ZnO seed layers was also reported to be another factor which affects the ZnO NWs' orientation [22]. However, in the present paper, it is found that the texture of ZnO seed layers does not affect the alignment. The XRD data for the seed layers with different thickness are shown in Figure 9. The ZnO seed layers with thickness under 10 nm do not show any reflection peak due to ultra-thin thickness. On the other hand, 10-, 20-, and 50-nm-thick seed layers appear the same diffraction peaks, indicating that the seed layers deposited at the same condition have the same texture. So, we suggest that the seed layers with different small thickness exhibit almost the same texture and do not have the major change with increase of thickness. Given the analysis above, we suggest that the texture of the ZnO seeds does not directly determine the ZnO NWs orientation in our experiments.

**Figure 10:**
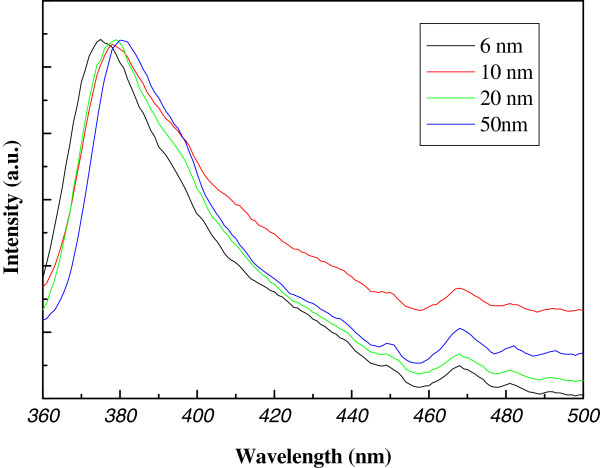
**The PL micrographs of ZnO NWs.** PL micrographs of ZnO NWs grown on pre-coated glass substrate with different thickness (from top to bottom, 2, 4, 6, 8,10, 20, and 50 nm).

PL spectroscopy is an effective technique for evaluating the optical properties and defects of semiconductor materials. Figure 10 shows typical room-temperature PL spectra of the ZnO NWs grown on glass substrates with different seed thickness. The PL spectra from all samples exhibit the same profile with a dominant emission peak centered at 383 nm, which corresponds to the ultraviolet emission of ZnO with a band gap of 3.24 eV [23]. In addition to the UV emission, two weak emissions at 450 and 468 nm also can be observed for the as-grown samples. The weak peaks in the blue-green band result from an electronic transition from the level of the ionized oxygen vacancies to the valence band [24]. We can see clearly that no obvious change of PL spectroscopy is occurred as the increase of seed thickness, which means that there is no relation between crystal defects and seed thickness.

## Implications of the hypothesis

We demonstrate that the growth of the ZnO NWs on ultra-thin seed layers is strongly influenced by the substrates and thickness of the seed films. NWs could be obtained on the smooth substrates covered with seed layers whose thickness is larger than 4 nm and have good alignment when roughness of the seed layers is also suitable. Besides, it is found that the thickness of the seed layers affects fluctuation amplitude and frequency of the roughness, which affects the alignment of the resulting NWs in succession. However, the crystal defects were influenced greatly by substrates instead of seed layers. The research provides prospect for preparation of the ZnO NWs on thin seed layers.

## Competing interests

The authors declare that they have no competing interests.

## Authors’ contributions

JND guided the work of this paper and revised the manuscript. YBL carried out the experiments and drafted the manuscript. CBT participated in the design of the experiments. NYY participated in the design of the studies and revised the manuscript. All authors read and approved the final manuscript.

## Authors’ information

JND is a professor at the Center for Low-Dimensional Materials, Micro-Nano Devices and System, Changzhou University, Changzhou 213164, China and at Jiangsu Key Laboratory for Solar Cell Materials and Technology, Changzhou, 213164, China. YBL and CBT are both post-graduates at the Center for Low-Dimensional Materials, Micro-Nano Devices and System, Changzhou University, Changzhou 213164, China. NYY is a professor at the Center for Low-Dimensional Materials, Micro-Nano Devices and System, Changzhou University, Changzhou 213164, China.
